# Structure, Intrinsic properties and Vibrational Spectra of Pr(Mg_1/2_Sn_1/2_)O_3_ Ceramic Crystal

**DOI:** 10.1038/s41598-017-13445-6

**Published:** 2017-10-17

**Authors:** Hengyang Qiao, Haiqing Sun, Jianzhu Li, Huiling Chen, Chao Xing, Jun Yang, Helei Dong, Jing Wang, Xunqian Yin, Ze-Ming Qi, Feng Shi

**Affiliations:** 1 School of Material Science & Engineering, Shandong University of Science and Technology, Qingdao, 266590 P.R. China; 2 Science and Technology on Electronic Test & Measurement Laboratory, North University of China, Taiyuan, 030051 P. R. China; 3 National Synchrotron Radiation Laboratory, University of Science and Technology of China, Hefei, 230029 P. R. China; 4 State Key Laboratory of High Performance Ceramics and Superfine Microstructure, Shanghai, 200050 P. R. China

## Abstract

Pr(Mg_1/2_Sn_1/2_)O_3_ (PMS) ceramic was prepared through a conventional solid-state reaction method. Crystal structure was investigated through X-ray diffraction (XRD), which certificates that the main phase is PMS with monoclinic *P2*_1_/*n1* symmetry. Lattice vibrational modes were obtained through Raman scattering spectroscopy and Fourier transform far-infrared reflection spectroscopy. The Raman spectrum active modes were assigned and illustrated, respectively, and then fitted with Lorentzian function. The four modes within the range of 110–200 cm^−1^ are derived from the *F*_*2g*_ vibrations (A-site cations), and the other three modes (300–430 cm^−1^) are derived from the *F*_*2g*_ vibrations (B-site cations).The mode with highest frequency above 650 cm^−1^ is attributed to *A*_*1g*_-like mode that corresponds to the symmetric breathing of oxygen octahedral. The far-infrared spectrum with seven infrared active modes was fitted using four-parameter semi-quantum models to calculate intrinsic properties (permittivity and loss). *F*_*2u*_^(2)^ yielded the greatest contribution to dielectric constant and loss, which is mainly performed as the inverted translational vibration of Pr-MgO_6_ octahedron.

## Introduction

In recent years, the mobile communication develops to high reliability with small size, then higher requirements are put forward for microwave dielectric ceramics (MWDCs). Thanks to the suitable permittivities (ε), high-quality factors (Q), and near-zero temperature coefficient of resonant frequency (τ_*f*_), *A*(*B*’_*1/2*_ *B*’’_*1/2*_)*O*_*3*_-type MWDCs are widely applied in resonators and filters. Rare-earth based MWDCs with double perovskites structure have been considered as potential candidate to substitute expensive complex perovskite MWDCs like Ba(Mg_1/3_Ta_2/3_)O_3_, which have been applied in commerce. Among these materials, Pr(Mg_1/2_Sn_1/2_)O_3_ (PMS) ceramics is promising with excellent properties in high frequency band of microwave frequency region.

Recently, more and more rare-earth based MWDCs, such as La(Mg_1/2_Ti_1/2_)O_3_, La(Zn_1/2_Ti_1/2_)O_3_ and La(Mg_1/2_Sn_1/2_)O_3_ had been widely studied^1–4^. The research of these ceramic materials mainly focuses on the basic structures^5,6^, lower the sintering temperatures^7,8^ and add additives to improve dielectric properties^9^. For example, crystal structures, dielectric properties and vibrational spectra of La(Mg_1/2_Sn_1/2_)O_3_ and La(Mg_1/2_Ti_1/2_)O_3_ ceramics were studied by Babu *et al*.^10,11^, which confirmed that these ceramics are the B-site ordered monoclinic crystal with space group *P2*_1_ */n1*. Subsequently, Chen *et al*. studied La(Mg_1/2_Sn_1/2_)O_3_ and Nd(Mg_1/2_Sn_1/2_)O_3_ ceramic doped with a variety of materials such as ZnO-B_2_O_3_-SiO_2_^1^, B_2_O_3_^12,13^, Yb_2_O^14^, ZnO^15^, and V_2_O_5_^16^.

There are many researches on praseodymium based materials, for example, to study the GMR properties of praseodymium based perovskites Pr_0.7_(Sr, Ca)_0.3_Mn_1−x_Al_x_O_3_ and Pr_0.5_Sr_0.5_Mn_1−x_Al_x_O_3_^17^, to study the catalytic properties of Pr_0.4_Ba_0.4_Ce_0.2_SrNiO_4_^18^, and to study the relationship between thermal expansion and oxygen ion transport of A_1−a_A_a_’BO_3_ perovskite-type oxides^19^.

However, there is no research on praseodymium based perovskites MWDCs up to date, for example, Pr(Mg_1/2_Sn_1/2_)O_3_ (PMS). Especially, applying both Raman scattering spectroscopy and Fourier transform far-infrared reflection spectroscopy together to investigate the vibrator parameters and the intrinsic properties of PMS is still unknown.

Here, in this work, PMS ceramic was synthesized through a conventional solid-state sintering technique. X-ray diffraction, and vibrational spectra (Raman and FTIR spectroscopies) were determined. We also used the four-parameter semi-quantum (FPSQ) model to calculate the intrinsic properties (dielectric constant and loss), which were compared with the data calculated by the Clausius-Mosotti equation (molecular polarizabilities), as well as the relationship between the damping coefficient and the intrinsic dielectric loss. The effect of the IR modes to the dielectric properties were analyzed. The Kramers–Krönig (K–K) analysis was conducted to acquire the real and imaginary parts of the dielectric constant for examine the vibrator parameters of PMS ceramic. This work may provide a basis of future research on the crystal structures, phone modes and intrinsic properties of rare-based *A*(*B’*_*1/2*_ *B”*_*1/2*_)*O*_*3*_-type MWDCs.

## Experiment

Pr(Mg_1/2_Sn_1/2_)O_3_ ceramics was prepared using a conventional solid-state reaction method. Pr_2_O_3_, MgCO_3_,and SnO_2_ powders with purity of 99.9% were used as raw materials with stoichiometric Pr:Mg:Sn = 2:1:1 molar ratio in polyethylene jars with zirconia balls for 4 h, next dried, and then calcined at 1200 °C for 4 h. After re-milling for 4 h, these mixed powders were dried and pressed into discs of 15 mm × 1 mm, and at last, sintered at 1500 °C for 4 h. The surfaces of the samples were carefully polished using micron-scale Al_2_O_3_ powder, and they were rubbed off about 20 micrometers before testing X-ray diffraction, and vibrational spectra.

XRD was conducted using a Rigaku D/max-rB X-ray diffractometer with Cu-*K*_*α*_ incident source within the 10°–80° 2θ range (0.02°, 2θ step size, and 1 s per step). Raman scattering spectra was obtained at room temperature by using a Nexus 670 spectrometer equipped with a liquid-N_2_-cooled CCD detector and an Olympus BXL microscope (100 × and 20 × objectives). Measurements were obtained in back-scattering geometry by using a Pr:YVO_4_ laser at 514 nm line as the excitation source (10 mW). Accumulation times were typically 10 collections at an interval of 5 s, and the spectral resolution was greater than 2 cm^−1^. The FTIR spectra were obtained at room temperature by using a Bruker IFS 66 v FTIR spectrometer with a highly sensitive DTGS detector. The laser source was He–Ne.

## Results and Discussion

**Figure 1:**
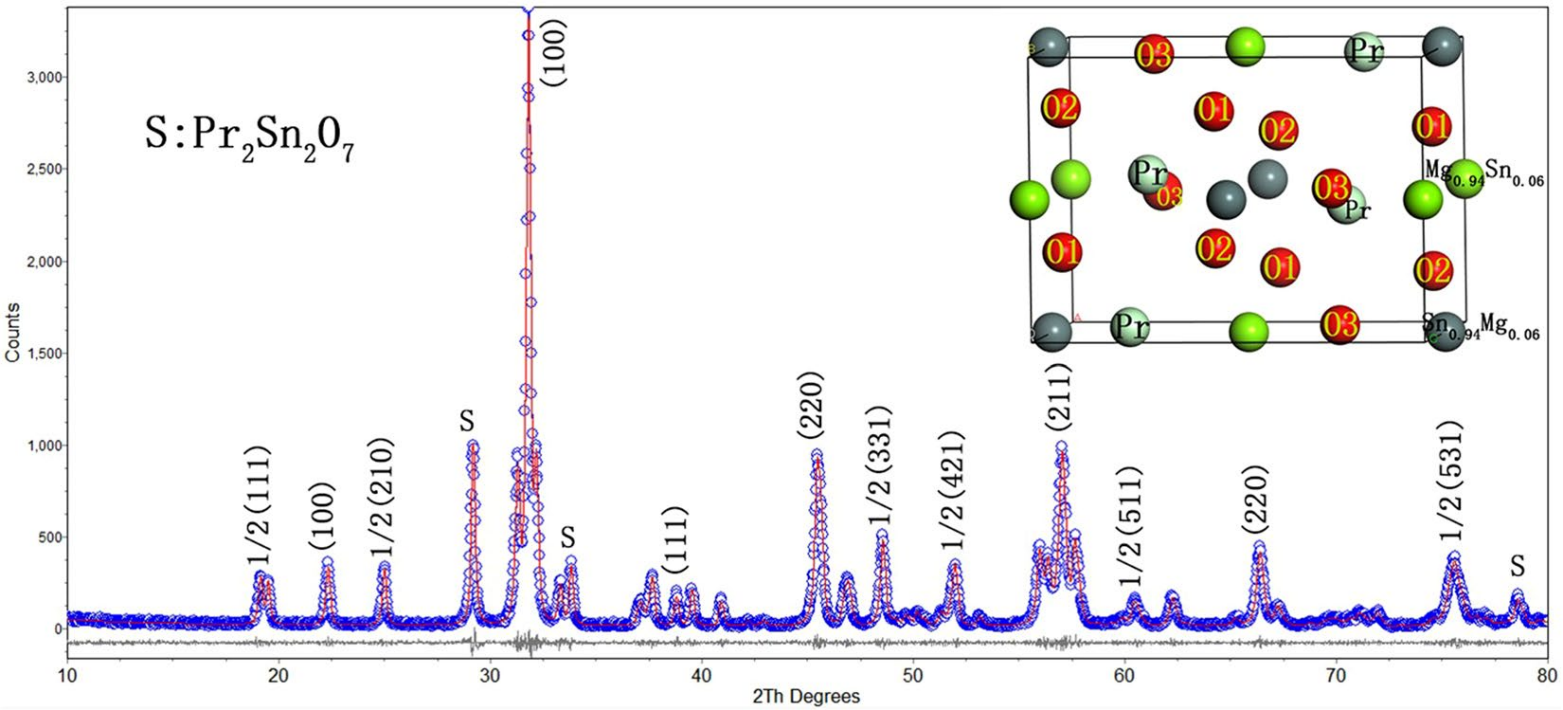
Observed XRD data (○ marks) and calculated (red line) from Rietveld refinements of Pr(Mg_1/2_Sn_1/2_)O_3_. The inset is the unit cell structure model of Pr(Mg_1/2_Sn_1/2_)O_3_ ceramics.

The XRD pattern after Rietveld refinement of the sample is presented in Fig. 1, which shows the main crystalline phase is PMS with double perovskite structure (monoclinic crystal system). The crystal structure model is shown in the top right corner of Fig. 1, whose crystal structure data are listed in Table 1. Some second phases, including Pr_2_Sn_2_O_7_ (JCPDS Card No. 13–0184, marked as S), are observed, due to the volatilization of magnesium.

**Table 1:** The Position of Each Element Atom of Pr(Mg_1/2_Sn_1/2_)O_3_ ceramics.

Atom	Site	x	y	z	Ion	Occupation
Pr	4e	0.4859	0.5535	0.2502	Pr^3+^	1
Mg1	2c	0	0.5	0	Mg^2+^	0.94
Mg2	2d	0.5	0	0	Mg^2+^	0.06
Sn1	2d	0.5	0	0	Sn^4+^	0.94
Sn2	2c	0	0.5	0	Sn^4+^	0.06
O1	4e	0.298	0.295	0.05	O^2−^	1
O2	4e	0.199	0.808	0.061	O^2−^	1
O3	4e	0.606	−0.032	0.257	O^2−^	1

A series of extra peaks was indexed by using half-integer Miller indices, such as 1/2(111), which correspond to superlattice reflection peaks. According to Glazer^20^, superlattice reflection peaks, with specific combinations of odd (o) and even (e) miller indices, can indicate the distortion type of crystal structure, such as octahedral in-phase tilting (ooe, oeo, eoo), octahedral anti-phase tilting (ooo, h + k + l > 3), the chemical order (ooo) and anti-parallel displacement of A-site cation (eoe, eeo, oee) etc. Here, o and e denote the odd number (o) and even number (e) in Miller index, respectively.

The extra superlattice peaks originate from the ordered arrangement of cations in the B-site. The diffraction peaks (1/2(210), 1/2(421) and 1/2(432)) are related to the cations in A-site, and the diffraction peaks (1/2(331) and 1/2(511)) are related to the anti-phase tilting of the octahedron. The existence of the diffraction peak 1/2(111) is the obvious evidence of the B-site cations 1:1 ordering. In addition, the splitting of diffraction peaks, such as peak (110) and peak (111), are observed, which indicates that the symmetry decreases.

For octahedral tilting, according to the following Eq. (1) ^21^:

1 $$t=\frac{{r}_{A}+{r}_{O}}{\sqrt{2}({r}_{B}+{r}_{O})}$$

**Figure 2:**
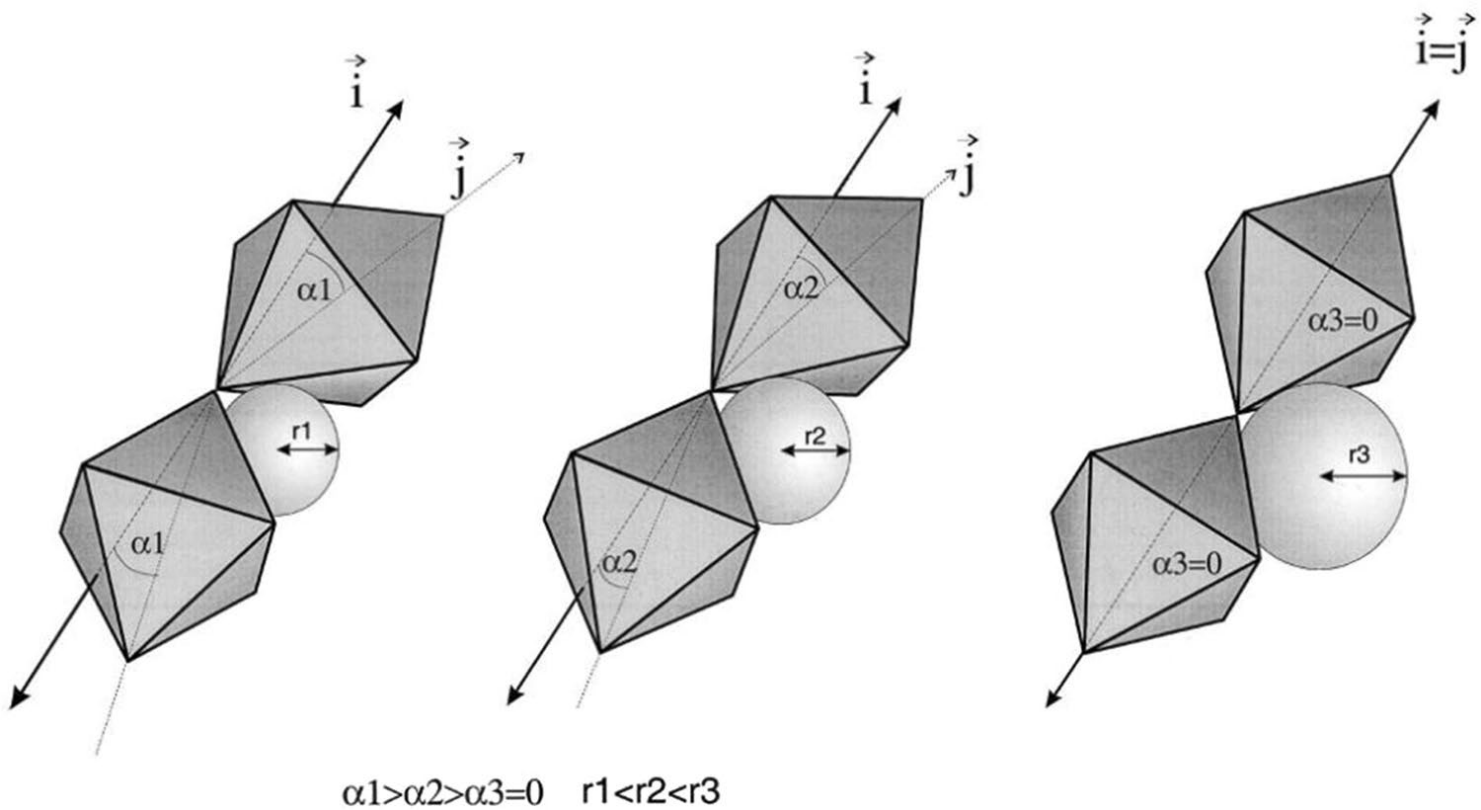
Schematic presentation of the correlation between tilt angles and A-site ionic radii (α1 > α2 > α3 = 0 r1 < r2 < r3)^22^.

The tolerance factor (*t*), where *r*_*A*_, *r*_*B*_ and *r*_*O*_ are the radii of the A-, B-site and O-ion, respectively. That is, the tolerance factor is positively related to the parameter of *r*_*A*_. The smaller of the tolerance factor (*t*), the larger the octahedral tilting. Figure 2 is the schematic presentation of the correlation between tilt angles and A-site ionic radii, referred by ref.^22^. Obviously, with the A-site ion radii decreases, the degree of octahedral tilting increases.

The Rietveld refinement results are listed in Table 2, which indicates *P2*_1_ */n*1 space group of the PMS sample.

**Table 2:** Crystallographic Data of Pr(Mg_1/2_Sn_1/2_)O_3_ Derived from Rietveld Refinement of XRD Data.

Formula	Pr(Mg_1/2_Sn_1/2_)O_3_
Crystal system	Monoclinic
Space group	*P121/n1*
a (Å)	5.5713191
b (Å)	5.7226529
c (Å)	7.9533907
Cell volume (Å^3^)	253.57549
Z	4
Software	Topas3
Radiation	Cu *K*α
Temperature (K)	293
Profile range in degree	10 ≤ 2 ≤ 80°
No. of data points	5501
*R*-factor:	
*R*_*p*_	7.02
*R*_*wp*_	10.33
*R*_*exp*_	9.01
*R-Bragg*	0.664

**Figure 3:**
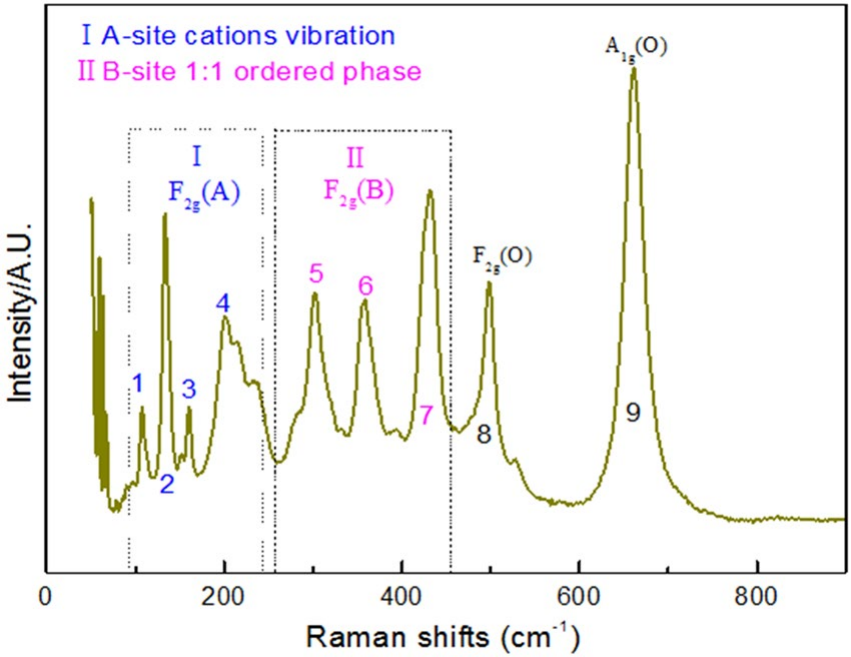
The Raman Spectra of PMS Ceramics in the range 50 cm^−1^ to 900 cm^−1^.

Figure 3 is the Raman scattering spectrum of PMS ceramic with Raman shifts from 50 cm^−1^ to 900 cm^−1^, which distinguishes 9 Raman active modes for the sample. According to group theory analysis, there are 24 (12*A*_1*g*_ + 12*B*_*1g*_)^13^ predicted Raman active modes for the phase-pure PMS. The Raman-active modes of ceramic with cubic $Fm\bar{{3}}m$ structure is (*A*_*1g*_ + 2*F*_*2g*_ + *E*_*g*_)^23^, and B-site 1:1 ordered structure can present four intense Raman active modes with *A*_*1g*_, 2*F*_*2g*_, and *E*_*g*_ symmetries. Here, we cannot identify all the 24 Raman active modes due to the mutual influence of the Raman active vibration modes and resolution of the measuring instrument.

When the excitation light interacts with the sample molecules, the energy exchange occurs after the collision of the photon with the sample molecules, and the photon obtains part of the energy from the sample molecules. So that the frequency of light is changed. Raman shift is the difference between the scattered light frequency and the incident light frequency.

The greater the molecular bond energy is, the greater the energy can get when the photon collides with the molecule, and the greater the Raman shift. The bond energy is mainly affected by the bond length, and the longer the bond length, the smaller the bond energy. In short, the longer the bond length, the lower the Raman shift, that is, Raman shifts are profoundly influenced by the bond lengths, and the bond length values of the Pr(Mg_1/2_Sn_1/2_)O_3_ ceramic were illustrated in Table 3.

**Table 3:** Bond length of the Pr(Mg_1/2_Sn_1/2_)O_3_ ceramics.

Bond type	D_-ave_(Å)
Pr-O	2.5482
Mg-O	2.0788
Sn-O	2.0788
O-O	2.9510

In Fig. 3, the modes of 1~4 within the range of 110–200 cm^−1^ are derived from *F*_2g_ vibration (A-site cations, part I). The modes within the range of 300–420 cm^−1^, *i.e*., modes 5, 6, and 7, correspond to the 1:1 ordered phase in B-sites (part II). The other two modes, 8 and 9, correspond to the vibration of oxygen atoms. The mode with the highest Raman shifts (above 650 cm^−1^) is attributed to the *A*_*1*g_-like mode, which corresponds to the symmetric breathing of oxygen octahedra because of the displacement of oxygen atoms along the Mg–O–Sn axis, whose frequency is primarily determined by the distances and bonding forces of Mg–O and Sn–O bonds^24^. The FWHM of the *A*_*1g*_ vibration mode is related to the B-site cation ordering^13^, *i.e*., B-O_6_ octahedron for O^2−^ ions in O1 and O2. The Raman spectrum with active modes can be fitted with Lorentzian function to obtain the full width at half maximum (FWHM); the values are listed in Table 4.

**Table 4:** The phonon modes parameters (Frequencies and FWHM values) from Raman spectra.

Modes	Frequency (cm^−1^)	FWHM (cm^−1^)
1 F_2g_(A)	107.71	2.32
2	132.99	1.14
3	159.15	2.19
4	201.57	4.56
5 F_2g_(A)	303.08	4.23
6	357.88	4.78
7	431.74	4.16
8 F_2g_(O)	497.69	3.94
9 A_1g_(O)	660.15	3.56

**Figure 4:**
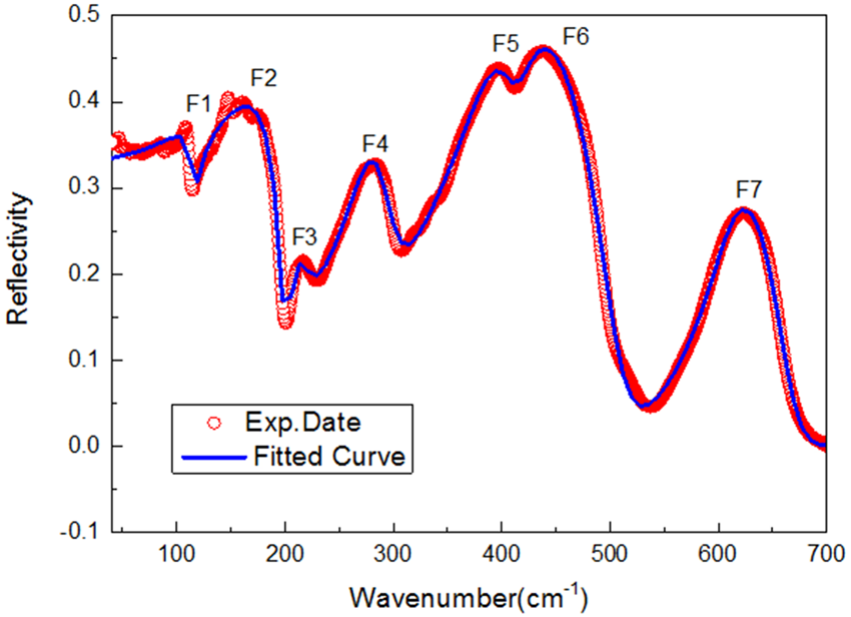
The FT-IR reflection spectrum of Pr(Mg_1/2_Sn_1/2_)O_3_.

The far-infrared reflection spectra of Pr(Mg_1/2_Sn_1/2_)O_3_ ceramics is shown in Fig. 4 with the wavenumber from 40 cm^−1^ to 700 cm^−1^. According to the group theory analysis^25^, the MWDCs with space group of *P2*_*1*_ */n1* have 33 infrared active vibration mode (*17A*_*u*_ + *16B*_*u*_), and the vibrational modes of *A*_*u*_ and *B*_*u*_ cannot split because the anisotropy tends to be balanced. Therefore, the number of the effective infrared active vibration mode of ceramic sample is 17^13^. In the far infrared reflectance spectrum, different vibrational modes correspond to different reflection bands. However, due to the instrument resolution and the mutual influence between vibration modes, it is difficult to distinguish all the reflection bands of 17 vibration modes, and only 7 reflection bands can be distinguished obviously, as shown in Fig. 4.

According to Eq. (3.2) in ref.^26^, the relationship between the dielectric constant and the frequency is inversely proportional. The curve is fitted by using the Lorentz FPSQ models to extract the vibrational frequencies of the IR modes. The vibrational frequencies of the IR modes were extracted by fitting the curve with Lorentz FPSQ models. These models describe the correlation between the complex permittivity (ε*) and vibrational modes of the ceramic materials, as follows Eq. (2):

2 $${{\rm{\varepsilon }}}^{\ast }({\rm{\omega }})={\rm{\varepsilon }}^{\prime} ({\rm{\omega }})-i{\rm{\varepsilon }}^{\prime\prime} ({\rm{\omega }})={\rm{\varepsilon }}\infty \prod _{j=1}^{n}\frac{{{\rm{\Omega }}}_{jLO}^{2}-{{\rm{\omega }}}^{2}+i{\rm{\omega }}{\gamma }_{jLO}}{{{\rm{\Omega }}}_{jTO}^{2}-{{\rm{\omega }}}^{2}+i{\rm{\omega }}{\gamma }_{jTO}}$$

(Four-parameter model)

3 $$R={|\frac{\sqrt{{\varepsilon }^{\ast }}-1}{\sqrt{{\varepsilon }^{\ast }}+1}|}^{2}$$

Where *ε*_∞_ refers to the optical permittivity, and *n* is the number of vibrational modes. *Ω*_*jTO*_, *γ*_*jTO*_, and *Ω*_*jLO*_, *γ*_*jLO*_ are the frequencies and damping factors of the *j*th transverse and longitude modes of vibration, respectively. The Fresnel equation [Eq. (3)] describes the relationship between the IR reflection spectrum and complex permittivity. Reflectivity (*R*) is an important parameter in the infrared reflectance spectrum. According to the Eq. (2) and the classical Kramers–Krönig (K-K) relation, the real part *ε*′ and the imaginary part *ε*′′ of the dielectric constant were calculated from the fitted spectra.

According to the results of four parameters fitting, the influence of each vibration mode on dielectric properties (permittivity *ε*_*j*_ and loss tan *δ*_*j*_/*ω*) is analyzed and discussed. The dielectric parameters can be calculated from Eqs (4) and (5), as follows:

4 $${{\rm{\varepsilon }}}_{j}=\frac{{\rm{\varepsilon }}\infty }{{{\Omega }}_{jTO}^{2}}\frac{\prod _{K}({{\Omega }}_{kLO}^{2}-{{\Omega }}_{jTO}^{2})}{\prod _{k\ne j}({{\Omega }}_{kTO}^{2}{-}{{\Omega }}_{jTO}^{2})}$$

5 $$\tan \,{\rm{\delta }}j/{\rm{\omega }}=\frac{{\rm{\varepsilon }}j\gamma /{{\rm{\Omega }}}_{jTO}^{2}}{{\rm{\varepsilon }}\infty +\sum _{j}{\rm{\varepsilon }}j}$$

The calculated *ε*_*j*_ and tan *δ*_*j*_/*ω* are also listed in Table 5. The vibrational modes at lower frequencies yield a larger contribution to the permittivity (*ε*_*j*_) and loss (tan *δ*_*j*_/*ω*). Thus, the dielectric properties are greatly affected by the modes involving more movements of heavier metal atoms. The first mode *F*_*1u*_^(1)^ can be assigned to the 1:1 ordered phase in B-sites. *F*_*2u*_^(2)^ also provides larger contribution to the dielectric constant and loss of a material than the other modes, which is mainly performed as the inverted translational vibration of Pr-MgO_6_ octahedron. Within the range of 200–500 cm^−1^ [*F*_*3u*_^(3)^, *F*_*4u*_^(4)^, *F*_*5u*_^(5)^ and *F*_*6u*_^(6)^], the vibrational modes correspond to the stretching vibrations of Mg–O–Sn. Within the range of 500–700 cm^−1^, *F*_*7u*_^(7)^ is regarded as the bending vibrations of Sn–O_6_. The permittivity and dielectric loss of PMS ceramics can be obtained by determining the sum of *ε*_*j*_ and tan *δ*_*j*_/*ω* of all the modes.

**Table 5:** Parameters of the IR-Active Modes for the Four-Parameter Model.

Number	modes	Ω_cal_ (cm^−1^)	Ω_jTO_ (cm^−1^)	γ_jTO_ (cm^−1^)	Ω_jLO_ (cm^−1^)	γ_jLO_ (cm^−1^)	Δε_j_	tanδ_j_/ω (10^−4^cm)
1	*F*_*1*_ *u* ^(*1*)^	108	114.79	17.88	115.56	12.58	0.24	0.236
2	*F*_*2*_ *u* ^(*2*)^	161	176.28	80.69	199.48	11.99	4.73	8.962
3	*F*_3_ *u* ^(*3*)^	216	206.00	21.09	220.10	46.53	0.41	0.147
4	*F*_4_ *u* ^(4)^	284	281.25	39.02	299.37	42.89	1.10	0.396
5	*F*_5_ *u* ^(5)^	398	387.85	68.23	411.66	35.07	2.28	0.755
6	*F*_6_ *u* ^(6)^	438	417.94	41.47	506.73	49.72	0.46	0.079
7	*F*_7_ *u* ^(7)^	623	606.14	54.47	665.02	36.90	0.40	0.043

*ε*_*∞*_ = 4.10.

The permittivity value (*ε*_*r*_) of IR fitting obtained by determining the sum of *ε*_*j*_ of all the modes is 13.72, and the calculated dielectric loss is $\sum \tan ({\rm{\delta }}j)/{\rm{\omega }}$ = 1.062 × l0^−3^.

The dielectric constant of PMS ceramics can also be calculated by Clausius-Mossi equation. According to the additivity rule of molecular polarizabilities, the molecular polarizability of the PMS ceramics can be obtained by adding the molecular polarizabilities of several simple substances, as shown in the following Eq. (6):

6 $$\alpha (\Pr (S{n}_{1/2}M{g}_{1/2}){O}_{3})=\alpha ({\Pr }^{3+})+\frac{1}{2}\alpha (S{n}^{4+})+\frac{1}{2}\alpha (M{g}^{2+})+3\alpha ({O}^{2+})$$

The single ion polarizability can be obtained from the research of Shannon R D^26^. Based on the molecular polarizability, the dielectric constant calculated by Clausius-Mossoi equation is 13.93, which is similar to the value of 13.72 by four-parameter fitting.

The quality factor *Q* is a basic measure of the energy loss in the microwave dielectric ceramic system and it is negatively correlated with the dielectric loss (*Q *=* 1/tan δ*). The dielectric loss mechanism includes the extrinsic loss associated with the preparation process and the intrinsic loss associated with the crystal structure.

The non-intrinsic loss can be effectively reduced by adjusting the preparation parameters. The intrinsic loss is sensitive to the micro-structure information, which is related to the vibration mode of the lattice. The intrinsic loss can be expressed as follows Eq. (7):

7 $$\tan \,\delta =(\frac{\gamma }{{\omega }_{T}^{2}}){\omega }_{0}$$

Where ω_T_ is the angular frequency of lattice vibration transverse optical mode, *γ* is the damping factor of the material. The classical radiation theory points out that the Full Width at Half Maximum (FWHM) of the Raman spectrum is closely related to the frequency and damping, and the damping coefficient can be calculated according to the following Eq. (8):

8 $$FWHM=\gamma \sqrt{{\gamma }^{2}+4{\omega }_{0}^{2}}/2{\omega }_{0}$$

Where *γ* is the damping factor and *ω*_0_ is the spectral center frequency. According to the Eq. (7) and Eq. (8), the second peak of the vibration mode *F*_*2g*_(*A*) in the Raman spectrum has the greatest influence on the intrinsic loss. The calculated intrinsic loss is 1.008 × 10^−3^, which is almost the same as the value 1.062 × l0^−3^ obtained by four-parameter fitting.

**Figure 5:**
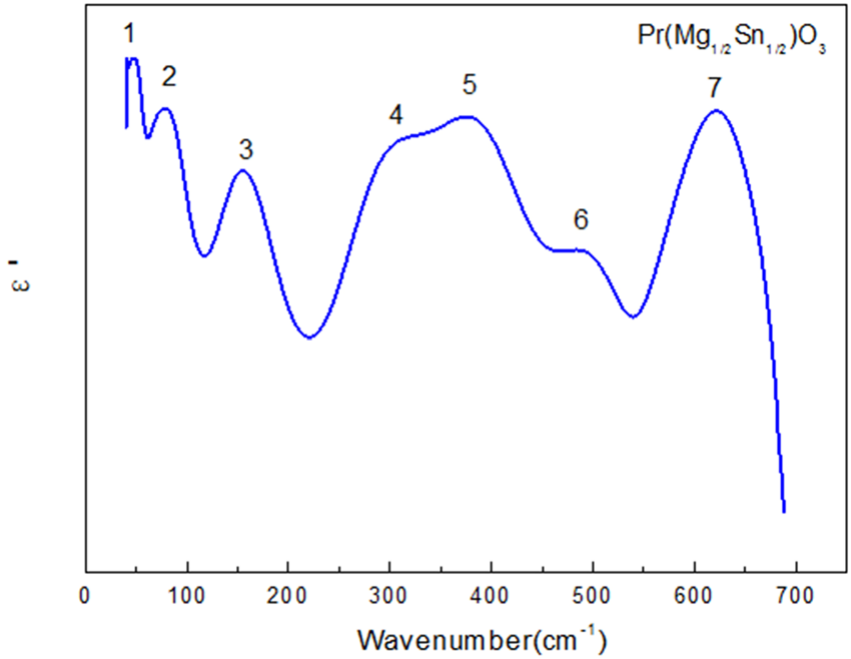
The real part of dielectric constants ε′(ω) calculated from K-K analysis for the Pr(Mg_1/2_Sn_1/2_)O_3_ ceramics.

The imaginary and real part of the PMS ceramics complex dielectric constant *ε*(*ω*) = *ε′*(*ω*) + *iε′′*(*ω*) can describe the characteristics of electromagnetic wave absorption of phonons. In Fig. 4, PMS ceramics infrared spectra were K-K conversion, and calculate the PMS ceramics complex dielectric constant $\varepsilon (\omega )=\varepsilon ^{\prime} (\omega )+i\varepsilon^{\prime\prime} (\omega )$. The imaginary part *ε*″(*ω*) and real part *ε*′(*ω*) of the dielectric constant are analyzed in detail. Figure 5 is the real part of dielectric constant *ε*′(*ω*) image of PMS ceramics after K-K conversion.

The real part *ε′*(*ω*), which is a function of wavelength, ranges from 40 cm^−1^ to 680 cm^−1^ (Fig. 5). For *ε′* of the PMS ceramics, seven peaks are shown in Fig. 5. Each peak is numbered from low to high frequency. The real part is the common dielectric constant and related to the dielectric properties. For ${\rm{\varepsilon }}^{\prime} =\frac{({{\rm{\varepsilon }}}_{s}-{{\rm{\varepsilon }}}_{\infty }){{\rm{\varepsilon }}}_{T}^{2}\gamma }{{\rm{\varepsilon }}[4{({{\rm{\varepsilon }}}_{T}-{\rm{\varepsilon }})}^{2}+{\gamma }^{2}]}$, the resonant frequency *ω*_*TO*_ of the vibrator can be determined from the real part of the dielectric constant when *ε′*(*ω*) reaches the maximum. Among these seven modes, No. 7 reaches the maximum value of the real part.

**Figure 6:**
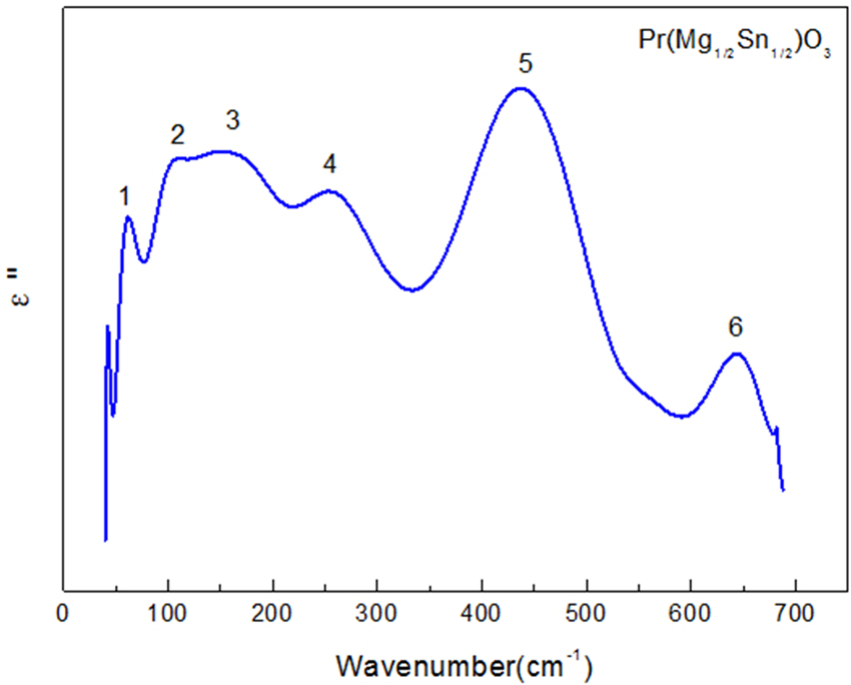
The imaginary part of dielectric constants ε′′(ω) calculated from K-K analysis for the Pr(Mg_1/2_Sn_1/2_)O_3_ ceramics.

As shown in Fig. 6, for PMS ceramics imaginary part of dielectric constant *ε′′*(*ω*) image can be clearly observed in 6 different modes of vibration. Each peak is numbered from low to high frequency, as in No. 1~6. The electromagnetic wave absorption characteristics of the vibrator are characterized by imaginary parts. These peaks are obtained through Lorentz fitting, and each peak corresponds to an IR-active mode.

## Conclusion

The Pr(Mg_1/2_Sn_1/2_)O_3_ ceramics were prepared through a conventional solid-state reaction method at 1500 °C for 4 h. The crystal structure was investigated through XRD, and the vibrational phone modes were investigated by Raman and FTIR reflection spectroscopies. The refinement plot of the XRD shows that the material exhibits a monoclinic double perovskite structure (*P2*_1_ */n*1). PMS is the main crystalline phase, which is accompanied by a small amount of Pr_2_Sn_2_O_7_ as the second phase. The Raman spectrum was obtained, and the highest Raman shifts mode above 660.9 cm^−1^ is attributed to the *A*_1*g*_(*O*)-like mode, which corresponds to the symmetric stretching of oxygen octahedron. The four modes within the range of 110–200 cm^−1^ are derived from the *F*_2*g*_ vibrations (A-site cations), and the other three modes (300–430 cm^−1^) are derived from the *F*_2*g*_ vibrations (B-site cations). The intrinsic loss (tan *δ*_*j*_/*ω*) calculated by FPSQ was 1.062 × l0^−3^, which agree well with the value of 1.008 × 10^−3^ calculated baed on damping coefficient and center frequency of optical mode. Through calculation and comparison, the second peak of the vibration mode *F*_*2g*_(*A*) in the Raman spectrum has the greatest influence on the intrinsic loss. We assigned the IR-active modes as *F*_1_ (108 cm^−1^), *F*_2_ (161 cm^−1^), *F*_3_ (216 cm^−1^), *F*_4_ (284 cm^−1^), *F*_5_ (398 cm^−1^), *F*_6_ (438 cm^−1^), and *F*_7_ (623 cm^−1^). Almost all of the atoms are involved in each IR vibrational mode, but each atom exhibits different effects. These data indicate that the vibrational modes at lower frequency (*F*_*2u*_^(2)^) provide larger contribution in terms of *ε*_*j*_ and tan*δ*_*j*_/*ω*. Thus, dielectric properties are mostly affected by modes involving more movements of heavier metal atoms, *i.e*., the inverted translational vibration of Pr-MgO_6_ octahedron. The permittivity value (*ε*_*r*_) of IR fitting obtained by determining the sum of *ε*_*j*_ of all the modes is 13.72, which agrees well with the permittivity value of 13.93 calculated by Clausius-Mossoi equation.
